# Antibiotic-resistant indicator bacteria in irrigation water: High prevalence of extended-spectrum beta-lactamase (ESBL)-producing *Escherichia coli*

**DOI:** 10.1371/journal.pone.0207857

**Published:** 2018-11-26

**Authors:** Maria-Theresia Gekenidis, Weihong Qi, Jörg Hummerjohann, Reinhard Zbinden, Fiona Walsh, David Drissner

**Affiliations:** 1 Microbiology of Plant Foods, Agroscope, Waedenswil, Switzerland; 2 Institute of Food, Nutrition and Health, ETH Zurich, Zurich, Switzerland; 3 Functional Genomics Center Zurich, ETH Zurich and University of Zurich, Zurich, Switzerland; 4 Microbiological Safety of Foods of Animal Origin, Agroscope, Liebefeld, Switzerland; 5 Swiss Expert Committee for Biosafety (SECB), Bern, Switzerland; 6 Department of Biology, Maynooth University, Maynooth, Ireland; 7 Department of Life Sciences, Albstadt-Sigmaringen University, Sigmaringen, Germany; University of Malaya Faculty of Medicine, MALAYSIA

## Abstract

Irrigation water is a major source of fresh produce contamination with undesired microorganisms including antibiotic-resistant bacteria (ARB), and contaminated fresh produce can transfer ARB to the consumer especially when consumed raw. Nevertheless, no legal guidelines exist so far regulating quality of irrigation water with respect to ARB. We therefore examined irrigation water from major vegetable growing areas for occurrence of antibiotic-resistant indicator bacteria *Escherichia coli* and *Enterococcus* spp., including extended-spectrum β-lactamase (ESBL)-producing *E*. *coli* and vancomycin-resistant *Enterococcus* spp. Occurrence of ARB strains was compared to total numbers of the respective species. We categorized water samples according to total numbers and found that categories with higher total *E*. *coli* or *Enterococcus* spp. numbers generally had an increased proportion of respective ARB-positive samples. We further detected high prevalence of ESBL-producing *E*. *coli* with eight positive samples of thirty-six (22%), while two presumptive vancomycin-resistant *Enterococcus* spp. were vancomycin-susceptible in confirmatory tests. In disk diffusion assays all ESBL-producing *E*. *coli* were multidrug-resistant (n = 21) and whole-genome sequencing of selected strains revealed a multitude of transmissible resistance genes (ARG), with *bla*_CTX-M-1_ (4 of 11) and *bla*_CTX-M-15_ (3 of 11) as the most frequent ESBL genes. Overall, the increased occurrence of indicator ARB with increased total indicator bacteria suggests that the latter might be a suitable estimate for presence of respective ARB strains. Finally, the high prevalence of ESBL-producing *E*. *coli* with transmissible ARG emphasizes the need to establish legal critical values and monitoring guidelines for ARB in irrigation water.

## Introduction

Antibiotic resistance worldwide costs thousands of lives every month and has been listed by the World Health Organization (WHO) among today’s biggest threats for global health, food safety, and development, since it threatens our ability to treat common infectious diseases [1, 2]. The antibiotic resistome has been defined as the sum of all genes directly or indirectly contributing to antibiotic resistance both in the clinics and the environment, straightening out the fact that antibiotic resistance is far from being confined to hospitals [3]. On the contrary, antibiotic resistance is an ancient phenomenon which has been shown to evolve in the absence of human activity [4]. Nevertheless, it is acknowledged that antibiotic resistance in the environment is on the rise due to selective pressure exerted through anthropogenic factors [5].

Of all environmental compartments, the aquatic ecosystems have been entitled as the main reservoir of antibiotic-resistant bacteria (ARB) [6]. The presence of numerous ARB and their resistance determinants in various surface waters has been well documented and has been linked frequently to nearby wastewater treatment plants (WWTP) [6–12]. Wastewater treatment plants have been described to enrich rather than reduce ARB and their resistance determinants before discharge into nearby rivers or lakes [7, 13]. A likely explanation for this enrichment is that WWTP combine several factors favoring exchange of antibiotic resistance genes (ARG) among bacteria and selection of resistant strains, namely high bacterial and nutrient density in the presence of residual antibiotics [14]. In a recent study, Farkas and coworkers found that multidrug-resistant (MDR) bacteria, i.e. bacteria with resistance to at least three antibiotic classes, were more prevalent in surface waters than in wastewater [15], again suggesting enrichment of these contaminants through WWTP before release into nearby surface waters.

Human exposure to these contaminants can occur through various routes. Apart from being used for recreational purposes, surface waters such as rivers or lakes are often used for irrigation of fresh produce [16]. In agricultural regions in which water is scarce, the use of reclaimed wastewaters for irrigation of fresh produce has become common practice [17–19]. Irrigation water is one of the major sources of fresh produce contamination with bacteria [20]. Especially irrigation through overhead sprinklers, a common irrigation technique in fresh produce cultivation, will maximize the probability of contamination of edible plant parts, i.e. the leaves [21]. Through its frequent raw consumption, fresh produce represents an ideal direct vector of microorganisms to the consumer. In the past decade it has been recognized that consumption of fresh produce exposes the consumer not only to potential foodborne pathogens, but also to ARB [22–25].

The diversity of ARB present on fresh produce is considerable [26]. However, only if these resistances are transmissible will they be of clinical relevance, as opposed to intrinsic resistance which cannot be easily spread within the bacterial community [27]. The latter is of low concern unless it is harbored by a pathogen, and expert rules defining the levels of antimicrobial resistance (that is, multidrug-resistant, extensively drug-resistant, and pandrug-resistant) ignore known intrinsic resistances [28].

Among the ARB harboring transmissible antibiotic resistances of utmost clinical relevance are extended-spectrum β-lactamase (ESBL)-producing *Enterobacteriaceae* including *Escherichia coli* and vancomycin-resistant *Enterococcus* spp. (VRE), which have both been listed among the top twelve serious drug-resistant threats by the Centers for Disease Control and Prevention (CDC) [29] and have been listed recently in the WHO priority list of antibiotic-resistant bacteria to guide research, discovery, and development of new antibiotics [30]. ESBL-producing *Enterobacteriaceae* are increasingly detected outside the hospital setting in the environment. More specifically, ESBL-producing strains have been isolated from different surface waters lately [31–34]. An extensive review by Guenther and colleagues on ESBL-producing *E*. *coli* in wildlife pointed out that first isolation of such strains in wild animals dates back to the year 2006 only, whereas other antibiotic-resistant *E*. *coli* had been isolated long before that, for the first time in the early 1980s and thereafter repeatedly all over the world [35]. This observation suggests a relatively recent spread of ESBL-producing *E*. *coli* into the environment. Of note, ESBL-producing *Enterobacteriaceae* have also been isolated from fresh produce [25]. As to the spread of VRE into the environment outside hospital settings, sporadic detection of VRE and ARG associated with vancomycin-resistance in various surface waters has been described [36–40], but there is no such report so far for Switzerland. Finally, apart from ARB the presence of a plethora of ARG and antibiotic residues has been described for different surface waters and groundwater and has been attributed to the widespread and excessive usage of antibiotics worldwide [7, 13, 41, 42].

The growing request for fresh and healthy food products in conjunction with the demand for sustainable water usage will result in increased future exploitation of surface waters, reclaimed, or even untreated wastewater for irrigation of fresh produce [43]. Although guidelines including critical values for indicator bacteria exist for use of surface water in fresh produce production in many countries, up to now compulsory guidelines addressing ARB in irrigation water are absent. The use of a few indicator organisms and where possible the relative quantification of selected resistance genes would largely facilitate the task of monitoring irrigation water quality with respect to ARB. *E*. *coli* and *Enterococcus* spp. are both classical indicators of fecal contamination routinely used in assessing microbiological quality of water as well as foods [44–46]. Both species have also been used as indicators for monitoring antibiotic resistance in food products [47], and they are both key players in the spread of antibiotic resistance [48–51], including strains of utmost clinical relevance [29]. As to relevant resistance determinants, as mentioned earlier ARG are of concern when they can spread within bacterial communities through horizontal gene transfer (HGT), e.g. via transmissible plasmids [52]. When observing phenotypic resistance in bacterial isolates, it is therefore important to determine the underlying resistance determinant in order to elucidate whether the observed resistance is of clinical relevance. The present study aimed at quantifying indicator generic bacteria *E*. *coli* and *Enterococcus* spp. in irrigation water from different vegetable growing areas and characterizing antibiotic-resistant strains thereof including their underlying resistance determinants, with a focus on ESBL-producing *E*. *coli* and VRE.

## Materials and methods

If not specified otherwise, material was purchased from Sigma-Aldrich (St. Louis, USA). No specific permission for sampling at the locations mentioned was required. Samples were taken within the Swiss Antibiotic Resistance Strategy (StAR) and the National Research Programme in collaboration with the Association of Vegetable Growers. We confirm that the field studies did not involve endangered or protected species.

### Water sampling and bacterial culture preparation

Water applied for irrigation of fresh produce from three major Swiss vegetable growing areas was sampled in July 2016. Sampled locations included Ammerswil, Baden-Rütihof, Birmenstorf, Brittnau, Buttwil, Hüttikon, Kirchleerau, Laufenburg, Muhen, Riehen, Seengen, Suhr, Therwil, Unterentfelden, Villigen, Wohlen, Wohlenschwil, Kerzers, Ins and Brüttelen. Samples originated from either groundwater or various types of surface water (rivers, water canals, creeks, ponds, spring water, and open and closed rain water tanks). The average distance from each sample collection was 42 km and the sampling sites were not related, that is, not connected by rain force with the exception of samples No. 3 and 4. All samples (1 l each, n = 36) were collected in sterile water sampling bottles (VWR, Radnor, USA), transported at approximately 8°C, and processed within 10 h.

To determine aerobic mesophilic counts, ten-fold dilution series and 100 μl of undiluted water sample were plated on PCA in duplicate. For enumeration of generic *E*. *coli* and *Enterococcus* spp., 100 ml were concentrated on nitrocellulose filters (0.22 μm pore size, EMD Millipore, Billerica, USA) in duplicate and transferred to either CHROMagar or mEA. After enumeration of generic *E*. *coli* and *Enterococcus* spp., filters from CHROMagar or mEA were placed on tryptic soy agar (TSA, 4 h, 37°C) and then transferred for enrichment into 10 ml EE broth or BPW, respectively (24 h, 37°C). Target ARB were then cultured by streaking 10 μl of enrichment broth onto the respective antibiotic-containing selective media.

### Bacterial culture conditions

The following media were used for bacterial cultivation: plate count agar (PCA) for determination of aerobic mesophilic count (AMC); CHROMagar *E*. *coli* (CHROMagar, Paris, France) and ready-to-use Brilliance ESBL plates (Oxoid Ltd., Hampshire, UK) for *E*. *coli*; m-Enterococcus agar (mEA) and ready-to-use Brilliance VRE plates (Oxoid Ltd.) for isolation of *Enterococcus* spp.

While AMC and generic *E*. *coli* and *Enterococcus* spp. were determined by direct incubation on PCA, CHROMagar, or mEA, respectively, antibiotic-resistant *E*. *coli* and *Enterococcus* spp. were cultured after enrichment. EE broth Mossel (Beckton Dickinson, Franklin Lakes, USA) was used for enrichment of *E*. *coli*, and buffered peptone water (BPW, 10.0 g of peptone, 5.0 g of NaCl, 3.5 g of anhydrous Na_2_HPO_4_, and 1.5 g of KH_2_PO_4_ per 1 l of water, pH 7.0) for enrichment of *Enterococcus* spp. After enrichment, ARB were cultured on the respective antibiotic-containing media: Additionally to commercial ESBL and VRE plates, CHROMagar and mEA plates supplemented with antibiotics were used. CHROMagar was supplemented with either ampicillin (AM, 100 mg/l), kanamycin (K, 16 mg/l), ciprofloxacin (CIP, 1 mg/l), or ceftazidime (CAZ, 8 mg/l), while mEA was supplemented with either erythromycin (ERY, 4 mg/l) or ciprofloxacin (1 mg/l). PCA plates were incubated at 30°C for 72 h; CHROMagar plates at 37°C for 24 h; mEA, VRE, and ESBL plates at 37°C for 48 h. Enrichment broths were incubated at 37°C for 24 h. All media were incubated under aerobic conditions.

### MALDI biotyping

Representative colonies were identified by MALDI biotyping by direct smearing as described previously [53] with a microflex LT MALDI-TOF mass spectrometer (Bruker Daltonics, Bremen, Germany) and the associated MALDI biotyper RTC Software (Version 3.1). All colonies displaying the typical blue coloration from filters incubated on CHROMagar were confirmed to be *E*. *coli*. Colonies identified from filters incubated on mEA were almost exclusively *Enterococcus* spp. (115 of 117, 98%), as was expected from previous findings [54]. Therefore, all colonies enumerated on mEA were designated as *Enterococcus* spp.

### Antibiotic susceptibility tests

*E*. *coli* and *Enterococcus* spp. isolated on either ESBL or VRE plates were screened for antibiotic resistances by disk diffusion assays. *E*. *coli* and *Enterococcus* spp. were tested against 32 or 11 clinically relevant antibiotics, respectively. After subculturing each strains on Columbia agar with 5% sheep blood (BioMérieux, Marcy l’Etoile, France) at 37°C and 7.5% CO_2_, disk diffusion assays were performed according to the European Committee of Antimicrobial Susceptibility Testing (EUCAST) guidelines from 2012 [55]. Briefly, bacterial suspensions of a turbidity equal to 0.5 McFarland was produced in 0.9% saline and were streaked on Mueller Hinton E (MHE) agar (Beckton Dickinson). Antibiotic disks were applied (i2a, Montpellier, France) and the plates were incubated at 35°C for 18 h ± 2 h or 24 h (*E*. *coli* or *Enterococcus* spp., respectively). Finally, inhibition zone measurement was performed using a Sirscan instrument (i2a) [56] followed by manual on-screen correction when needed. To determine antibiotic susceptibility, epidemiological cutoff (ECOFF) values were used according to EFSA recommendations for epidemiological screenings [57]. When ECOFF values were absent, clinical breakpoints were used (cefpodoxime and fosfomycin for *E*. *coli*), and when no value was defined in EUCAST guidelines, Clinical and Laboratory Standards Institute (CLSI) breakpoints were applied (colistin, minocycline, kanamycin, sulfonamide, tetracycline, temocillin, and cefalotin for *E*. *coli*; gentamicin high concentration, erythromycin, tetracycline, and chloramphenicol for *Enterococcus* spp.). Species with intrinsic resistances as defined by EUCAST expert rules were considered resistant (that is, erythromycin-resistance in *Enterococcus faecalis*) [58].

Presumptive ESBL-producing strains were confirmed by inoculating MHE agar as well as MHE agar containing cloxacillin (Axon Lab AG, Baden, Switzerland) and applying six antibiotic disks (cefoxitin with or without cloxacillin, cefotaxime with or without clavulanic acid, and ceftazidime with or without clavulanic acid) [59]. Plates were then incubated at 35°C for 18 h ± 2 h and inhibition zones were evaluated. Confirmation of presumptive VRE strains was performed using E-TEST Antimicrobial Resistance Detection strips (BioMérieux) on MHE agar inoculated with the test strain from a normalized bacterial suspension (0.5 McFarland). After incubation at 35°C for 24 h, the inhibitory concentration was read out.

### Phylogenetic groups

*E*. *coli* phylogenetic groups (PG) were determined as described by Clermont and colleagues [60] by quadruplex PCR amplification. PCR was performed with custom-synthesized primers (Microsynth, Balgach, Switzerland) and a DreamTaq hot start PCR master mix (Thermo Fisher Scientific, Waltham, USA). Upon band visualization on a TBE gel (2% agarose, 35 min, 100 V), strains displaying ambiguous patterns were subjected to confirmatory C- or E-PCR.

### DNA extraction, sequencing, and bioinformatics

Genomic DNA was extracted from eleven selected ESBL strains from different irrigation water samples covering the observed variety of phylogenetic group and antibiotic resistance with the commercial kit GenElute Bacterial Genomic DNA (Sigma-Aldrich). DNA was custom-sequenced using paired-end Illumina (HiSeq4000, 2 × 150 bp, 483 bp average insert size) at GATC Biotech (Konstanz, Germany). Data was processed using CLC Genomics Workbench Version 10.0 (Qiagen, Venlo, Netherlands) and resulting contigs were screened for genes of interest using online tools from the Center for Genomic Epidemiology (CGE): MLST 1.8, SerotypeFinder 1.1, VirulenceFinder 1.5, and ResFinder 3.0 [61–64].

To enrich plasmid DNA, extracts were produced using commercial PureYield Plasmid Maxiprep System (Promega, Fitchburg, USA). DNA was sequenced using a Pacific Biosciences RSII instrument at the Functional Genomics Center Zurich (8-kb insert library, P6/C4 chemistry). Four to five plasmid extracts were pooled per library. After size selection at 5 kb using a BluePippin (Sage Science, Beverly, USA) 360 min movies were recorded from each cell. Raw reads were assembled using Canu (version 1.5) [65]. Hybrid assemblies were produced (1) as polished Canu contigs (pilon, version 1.22) scaffolded with paired-end Illumina reads (SGA scaffolder, version v0.10.15) [66, 67] or (2) as assembled Illumina contigs scaffolded with Canu contigs and unassembled PacBio subreads (SPAdes, version 3.10.1) [68] to draw more robust conclusions from the two complementary approaches. Resulting scaffolds were screened for genes of interest as mentioned above. Additionally, the online tool Multiple Antibiotic Resistance Annotator (MARA) was used to identify mobile genetic elements in the genetic environment of the detected ARG [69].

### Statistical analysis

Fisher’s exact test was applied to determine statistical significance of the observed increased frequency of ARB-positive irrigation water samples with increasing generic *E*. *coli* or *Enterococcus* spp. counts (*P* < 0.05).

## Results

### Aerobic mesophilic count and generic *E*. *coli* and *Enterococcus* spp

**Fig 1:**
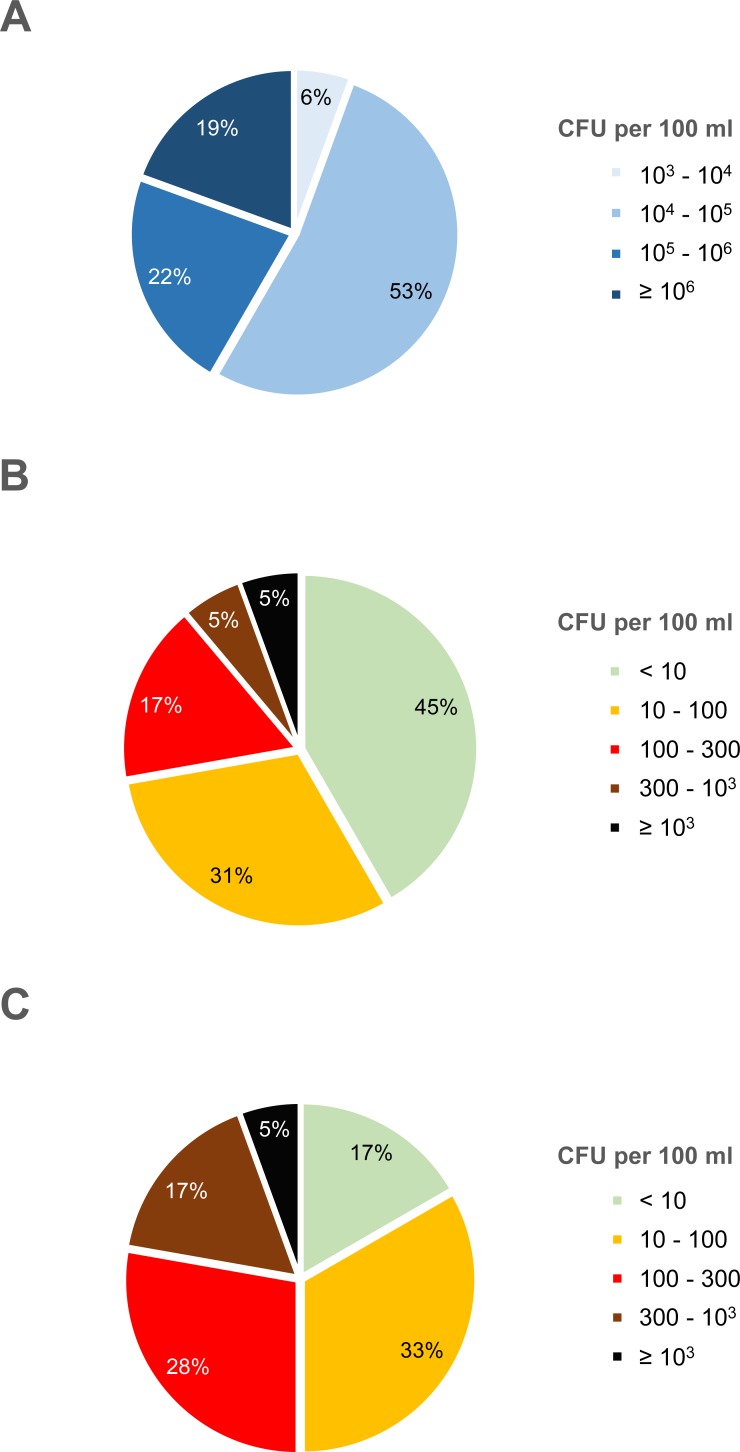
Aerobic mesophilic counts and generic *E*. *coli* and *Enterococcus* spp. counts in irrigation water. Colony forming units (CFU) per 100 ml of water are indicated. (A) Aerobic mesophilic counts. (B) *E*. *coli*. (C) *Enterococcus* spp. For generic *E*. *coli* and *Enterococcus* spp., the first category (≤ 9 CFU per 100 ml) comprises samples from below the detection limit (1 CFU per 200 ml) up to 9 CFU per 100 ml. The percentage of water samples (total n = 36) falling into each category is displayed.

Aerobic mesophilic bacterial counts (AMC) ranged from 1.0 × 10^3^ to 3.9 × 10^6^ CFU per 100 ml, with about half of the samples (19 of 36) lying between 10^4^ and 10^5^ CFU per 100 ml (Fig 1A). No correlation was observed between AMC and either *E*. *coli* or *Enterococcus* spp. counts, i.e. increased AMC did not correlate with increased *E*. *coli* or *Enterococcus* spp. counts (S1 Fig). *E*. *coli* ranged from undetectable (< 1 CFU per 200 ml) to 1.0 × 10^3^ CFU per 100 ml (Fig 1B). Almost half of the samples (16 of 36) had very low to undetectable *E*. *coli* counts (< 10 CFU per 100 ml). Two samples reached 1.0 × 10^3^ CFU per 100 ml. *Enterococcus* spp. ranged from below the limit of detection (< 1 CFU per 200 ml) to 1.2 × 10^3^ CFU per 100 ml (Fig 1C). *Enterococcus* spp. counts ranged from undetectable to at least 1.0 × 10^3^ CFU per 100 ml as was observed for *E*. *coli*, however, more samples fell into the intermediate categories. About one in five water samples (22%) contained *Enterococcus* spp. above 300 CFU per 100 ml.

### Antibiotic-resistant *E*. *coli* and *Enterococcus* spp

Antibiotic-resistant *E*. *coli* and *Enterococcus* spp. were isolated on either CHROMagar or mEA, respectively, containing selected antibiotics. In six samples, no ARB *E*. *coli* or *Enterococcus* spp. could be isolated on any of the tested antibiotics (samples 6, 7, 13, 19, 20, and 25; Table 1). In three of these samples no generic *E*. *coli* but generic *Enterococcus* spp. were detected (samples 19, 20, and 25; grey and white in Table 1, respectively), and in one sample neither generic *E*. *coli* nor generic *Enterococcus* spp. were detected (sample 13, grey in Table 1).

**Table 1:** Antibiotic-resistant *E*. *coli* and *Enterococcus* spp. isolated from irrigation water of different Swiss vegetable growing areas.

*no*.	*origin*	*AM*	*K*	*CIP*	*CAZ*	*ESBL*	*ERY*	*CIP*	*VRE*	*sequencing*
1	canal A									
2	canal B					√				H2, H6, H17
3	canal C					√				H10, H25
4	river A					√				H22
5	pond									
6	rainwater tank (o)									
7	rainwater tank (c)									
8	rainwater tank (c)									
9	pond									
10	pond									
11	pond									
12	pond									
13	groundwater									
14	groundwater									
15	pond									
16	rainwater tank (o)									
17	rainwater tank (o)									
18	pond								**×**	
19	rainwater tank (c)									
20	rainwater tank (c)									
21	rainwater tank (c)									
22	spring water					√				H30
23	pond								**×**	
24	rainwater tank (o)					√				H44
25	groundwater									
26	pond									
27	rainwater tank (c)									
28	rainwater tank (c)									
29	pond									
30	pond									
31	rainwater tank (c)					√				H38
32	river B					√				H40
33	pond									
34	creek									
35	creek									
36	canal D					√				H45

Grey: no generic *E*. *coli* or *Enterococcus* spp. detected on CHROMagar or mEA without antibiotics; white: only generic *E*. *coli* or *Enterococcus* spp. detected; blue: *E*. *coli* detected on CHROMagar containing the respective antibiotic or ESBL agar; pink: *Enterococcus* spp. detected on mEA containing the respective antibiotic or VRE agar. AM: ampicillin; K: kanamycin; CIP: ciprofloxacin; CAZ: ceftazidime; ESBL: commercial ESBL agar; ERY: erythromycin; VRE: commercial VRE agar; (c), closed; (o), open. √: ESBL-producing *E*. *coli* confirmed; **×**: vancomycin-resistant *E*. *faecalis* not confirmed. Sequencing: sequenced ESBL-producing *E*. *coli* (whole-cell and plasmid extracts).

**Fig 2:**
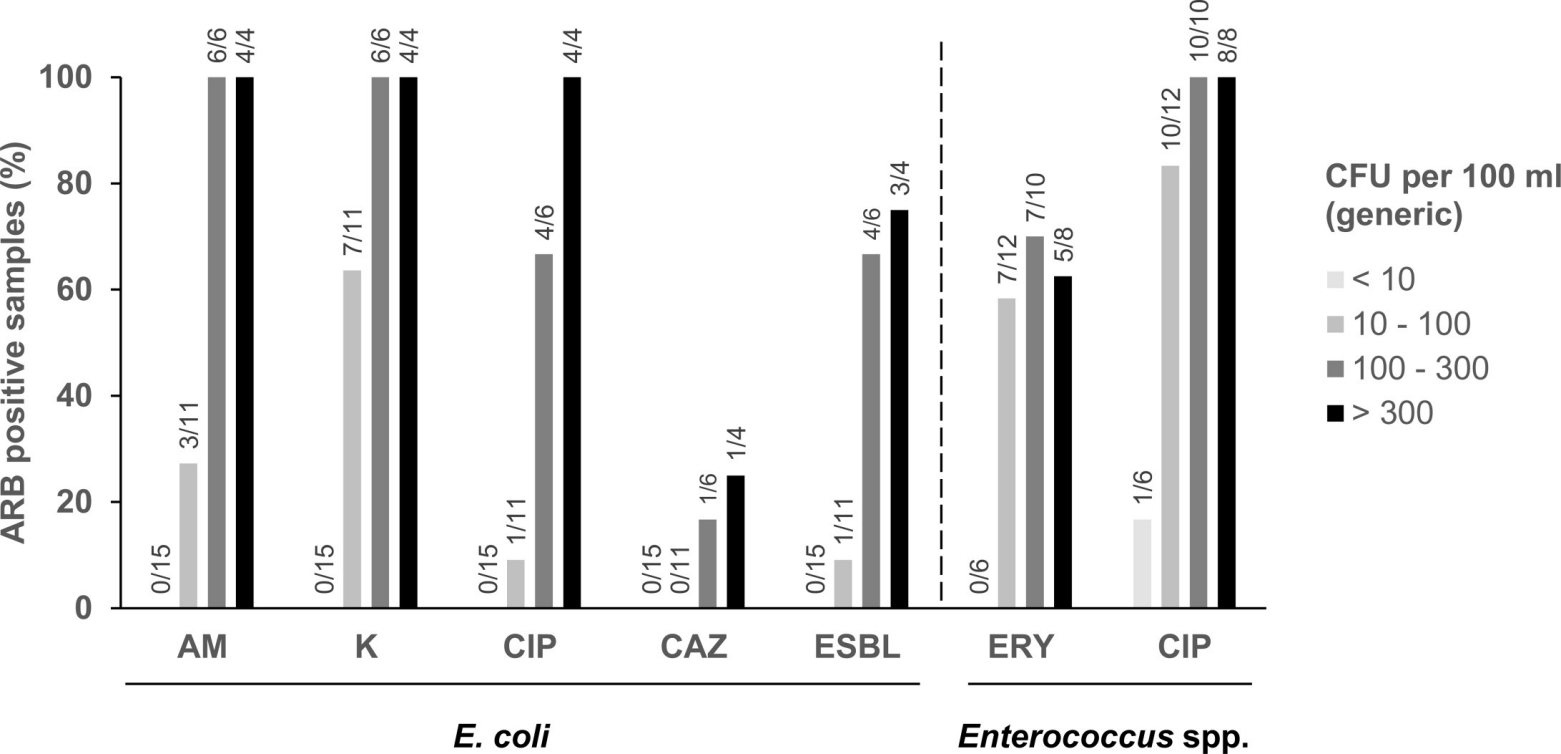
Generic *E*. *coli* or *Enterococcus* spp. counts and frequency of ARB-positive irrigation water samples. With increasing generic *E*. *coli* or *Enterococcus* spp. counts, water samples containing different ARB of the respective species were more frequent. AM, ampicillin; K, kanamycin; CIP, ciprofloxacin; CAZ, ceftazidime; ERY, erythromycin. Numbers on top of bars indicate number of positive to total water samples.

*E*. *coli* were most frequently isolated on kanamycin, followed by ampicillin, ciprofloxacin, and ceftazidime. Finally, presumptive ESBL-producing *E*. *coli* were cultured from eight of 36 water samples (22%), and all could be confirmed to be ESBL-producing *E*. *coli* in subsequent testing. The proportion of water samples containing *E*. *coli* growing on antibiotic-containing plates in dependence of generic *E*. *coli* content is shown in Fig 2. For all antibiotic plates, increased generic *E*. *coli* counts correlated with increased proportion of ARB *E*. *coli* positive samples. For instance, while no ESBL-producing *E*. *coli* were detected in samples with the lowest generic *E*. *coli* counts (< 10 CFU per 100 ml), proportion of ESBL-positive samples increased as generic *E*. *coli* counts increased (Fig 2). This increase was statistically significant with the only exception of CAZ-resistant *E*. *coli* (*P* < 0.0001 for AM-, K-, and CIP-resistant *E*. *coli*; *P* < 0.001 for ESBL-producing *E*. *coli* and CIP-resistant *Enterococcus* spp.; *P* < 0.05 for ERY-resistant *Enterococcus* spp.).

*Enterococcus* spp. were isolated on ciprofloxacin from 29 of the 36 samples (Table 1) and from about half the samples (19 of 36) on erythromycin. Two samples contained presumptive VRE *E*. *faecalis*. In a confirmatory E-TEST, however, they proved vancomycin-susceptible with minimal inhibitory concentrations of 1 mg or 4 mg per l (resistance cutoff: > 4 mg/l). As for *E*. *coli*, the proportion of water samples positive for *Enterococcus* spp. isolated on broad-spectrum antibiotic ciprofloxacin increased with increasing generic *Enterococcus* spp. counts (Fig 2): While only one of six samples with less than 10 CFU per 100 ml generic *Enterococcus* spp. was positive for such strains, more than three quarter of the samples with 10 to 100 CFU per 100 ml and all water samples with more than 100 CFU per 100 ml were positive (Fig 2). The proportion of samples positive for erythromycin-resistant *Enterococcus* spp. similarly increased with higher generic *Enterococcus* spp. counts (only exception: > 300 CFU per 100 ml generic *Enterococcus* spp.; Fig 2).

### Antibiograms of ESBL-producing *E*. *coli* and presumptive VRE

**Fig 3:**
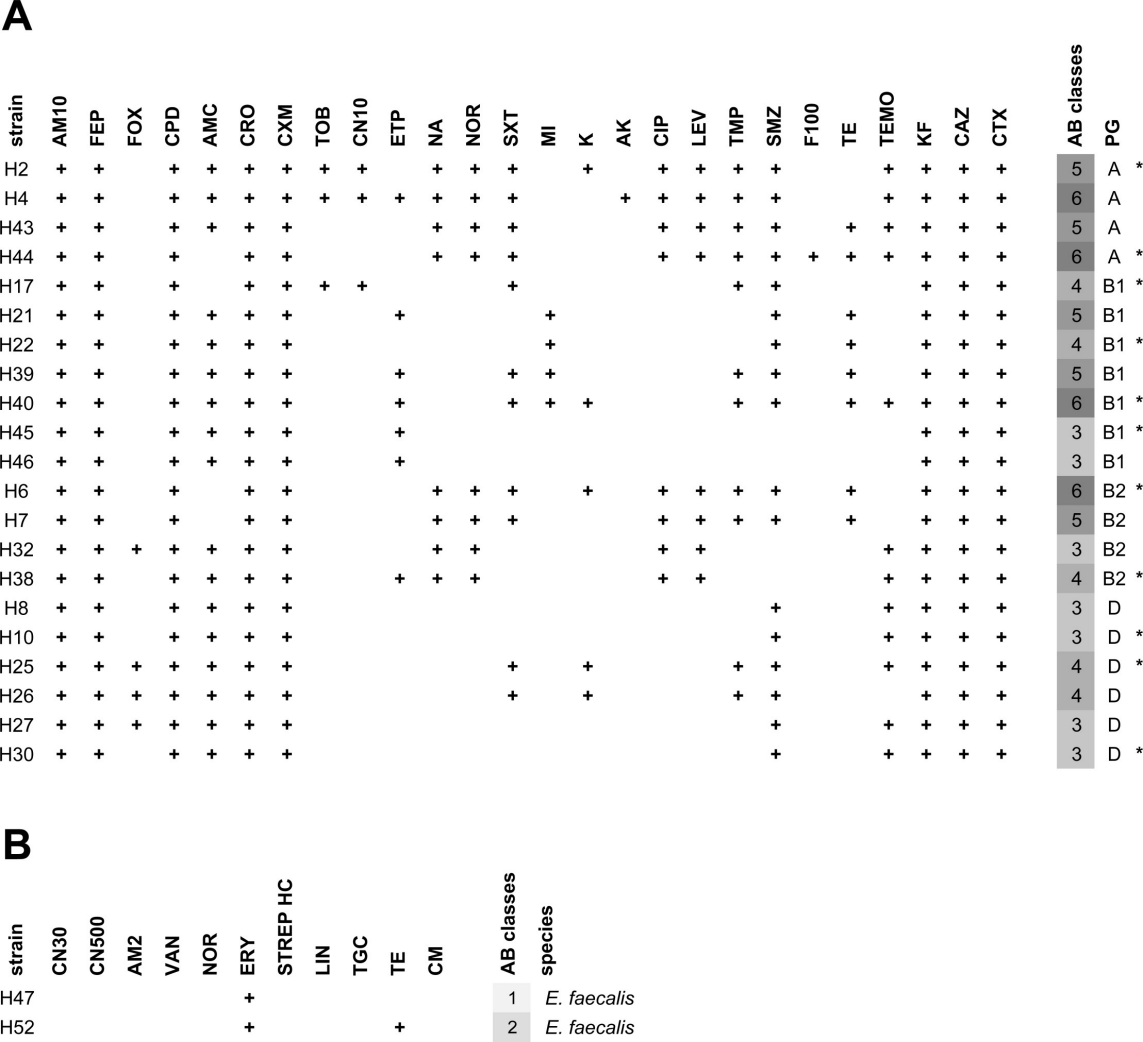
Antibiograms of ESBL-producing *E*. *coli* and presumptive VRE *E*. *faecalis* from irrigation water. *E*. *coli* and *E*. *faecalis* were tested against 32 or 11 clinically relevant antibiotics, respectively. Isolates were grouped by phylogenetic group. (A) Resistance profiles of ESBL-producing *E*. *coli*. AM10: ampicillin 10 μg; FEP: cefepime; FOX: cefoxitin; CPD: cefpodoxime; AMC: amoxicillin-clavulanic acid; CRO: ceftriaxone; CXM: cefuroxime; TOB: tobramycin; CN10: gentamicin 10 μg; ETP: ertapenem; NA: nalidixic acid; NOR: norfloxacin; SXT: trimethoprim-sulfamethoxazole; MI: minocycline; K: kanamycin; AK: amikacin; CIP: ciprofloxacin; LEV: levofloxacin; TMP: trimethoprim; SMZ: sulfonamide; F100: nitrofurantoin; TE: tetracycline; TEMO: temocillin; KF: cefalotin; CAZ: ceftazidime; CTX: cefotaxime; PG: phylogenetic group; AB classes: number of antibiotic classes. No resistance was observed to meropenem, piperacillin-tazobactam, colistin, tigecycline, fosfomycin, and imipenem. Asterisks (*) mark isolates used for subsequent plasmid extraction and sequencing. (B) Resistance profiles of presumptive VRE *E*. *faecalis*. CN30: gentamicin 30 μg; CN500: gentamicin 500 μg; AM2: ampicillin 2 μg; VAN: vancomycin; NOR: norfloxacin; ERY: erythromycin; STREP HC: streptomycin high concentration; LIN: linezolid; TGC: tigecycline; TE: tetracycline; CM: chloramphenicol.

For ESBL-producing *E*. *coli* and presumptive VRE, antibiotic resistance to clinically relevant antibiotics was determined in disk diffusion assays. All tested *E*. *coli* were resistant to ampicillin and the cephalosporins cefalothin (1^st^ generation), cefuroxime (2^nd^ generation), cefpodoxime, ceftriaxone, ceftazidime, and cefotaxime (3^rd^ generation), and cefepime (4^th^ generation) (Fig 3A). Further frequent resistances (more than half of the isolates) were detected to amoxicillin-clavulanic acid, sulfamethoxazole, and temocillin. Resistance to the carbapenem antibiotic ertapenem was detected in 7 of the 21 isolates. No resistance was observed in any of the tested strains to meropenem, imipenem, piperacillin-tazobactam, colistin, tigecycline, and fosfomycin. All isolates were MDR (resistant to at least three classes) and 9 of the 21 isolates were resistant to at least five antibiotic classes (Fig 3A). The two presumptive VRE strains identified by MALDI biotyping as *E*. *faecalis* carried intrinsic erythromycin-resistance and one was tetracycline-resistant (Fig 3B). In subsequent E-TEST none of the two strains proved vancomycin-resistant.

### ESBL-producing *E*. *coli*

#### Typing and virulence factors

Eleven ESBL-producing *E*. *coli* strains covering all ESBL positive irrigation water samples were sequenced. Assembled contigs and scaffolds were used for strain typing with online tools. Phylogenetic groups A, B1, B2, and D were detected, with group B1 being the most prevalent one (4 of 11 strains, Table 2). Multilocus sequence typing revealed that two ESBL-producing strains isolated from different water samples (H10 and H30 from water canal B and spring water, respectively) belonged to ST-68 and had all investigated characteristics in common including serotype, virulence factors, and antibiotic resistance profile and determinants. The remaining ESBL-producing strains belonged to different sequence types including one allele combination with not assigned a known sequence type (strain H45, Table 2). Serotypes could be determined for all except three strains, in which no O-antigen was detected. The three O-serogroups, which could not be determined based on sequencing data were analyzed by agglutination at the German Federal Institute for Risk Assessment (BfR, Berlin, Germany).

**Table 2:** Virulence factors detected in ESBL-producing *E*. *coli* from irritation water by whole-cell and plasmid DNA sequencing.

*ID*	*origin (PG*, *ST)*	previous isolation^*^	*serotype*	*virulence factors*
H2	canal B (A, ST-361) [77]	human, livestock, cheese, water, sewage	O9:H30	*gad*, *capU*
H6	canal B (B2, ST-1193) [78]	human, dog, livestock, water, sewage	O75:H5	*gad*, ***iha***, *sat*, *vat*, ***senB‡***, ***celb***
H17	canal B (B1, ST-1079) [79]	human, livestock, wild animals, lettuce, water, sewage	O6:H49	***gad*, *lpfA***
H10	canal C (D, ST-68) [80]	human, dog, cat, livestock, wild animals	O99:H6	*gad*, ***iss***, *lpfA*, *eilA*
H25	canal C (D, ST-38) [81, 82]	human, dog, livestock, wild animals, cheese, barley, water, sewage	**O153**:H30	*gad*, ***iss***, ***iha***, *eilA*, *sat*, *capU*, ***aap***, ***aar***, *aatA*, *aggA-D*, ***aggR***, *ORF3*, *ORF4*
H45	canal D (B1, n.d.†)	–	**O8**:H2	***gad***, *iss*, ***lpfA***,
H30	spring water (D, ST-68) [80]	human, dog, cat, livestock, wild animals	O99:H6	*gad*, ***iss***, *lpfA*, *eilA*
H22	river A (B1, ST-641) [83]	human, livestock, wild animals, celery, water, sewage	O159:H21	*gad*, ***iss***, ***lpfA***,
H40	river B (B1, ST-58) [83]	human, dog, livestock, wild animals, dairy, spinach, feed, water, sewage, soil	ONT:H37	*iss*, ***lpfA***, ***cba***, *cma*
H38	rainwater tank (c) (B2, ST-131) [77]	human, cat, dog, livestock, wild animals, dairy, water, sewage	O25:H4	***gad***, ***iss***, ***iha***, ***sat***
H44	rainwater tank (o) (A, ST-4981) [84]	human, livestock, flies	O89:H9	***gad***, *iss*

PG, phylogenetic group according to Clermont et al. [60]; ST, sequence type; (c), closed; (o), open; ONT, O not typable; bold **O**, determined by agglutination; *aap*, dispersin; *aatA*, dispersin transporter protein; *aar*, *aggR*-activated regulator; *aggA*, AAF/I major fimbrial subunit; *aggB*, AAF/I minor adhesion; *aggC/D*, usher/chaperone (AAF/I assembly unit); *aggR*, *araC* transcriptional activator; *capU*, hexosyltransferase homolog; *cba*, colicin B; *cma*, colicin M; *celb*, endonuclease colicin E2; *eilA*, *Salmonella* HilA homolog; *gad*, glutamate decarboxylase; *iha*, adherence protein; *iss*, increased serum survival; *lpfA*, long polar fimbriae; *ORF3*, isoprenoid biosynthesis; *ORF4*, putative isopentenyl-diphosphate delta-isomerase; *sat*, secreted autotransporter toxin; *senB*, plasmid-encoded enterotoxin; *vat*, vacuolating autotransporter toxin. All virulence factors covered the full length of the detected virulence gene. Bold virulence factors indicate 100% identity between query sequence and virulence gene sequence while slim virulence factors mark imperfect matches (> 98.5% identity).

† *adk-6*, *fumC-4*, *gyrB-14*, *icd-642*, *mdh-9*, *purA-7*, *recA-7*

‡ plasmid-encoded

* based on sequence type (http://enterobase.warwick.ac.uk/)

A multitude of virulence factors was detected, varying between strains in number and combination. Glutamate decarboxylase (*gad*) was present in all but one strain (10 of 11), followed by increased serum survival (*iss*) and long polar fimbriae (*lpfA*) detected in 8 and 6 of 11 strains, respectively (Table 2). The remaining virulence factors including the putative adherence protein *iha* were detected in one to three ESBL-producing strains. Strain H38 belonging to the globally spread MDR pandemic clone B2:ST-131 [70] contained apart from *gad*, *iss*, and *iha* the secreted autotransporter toxin *sat*. Strain H6 B2:ST-1193 harbored three toxin-encoding genes *sat*, the vacuolating autotransporter toxin *vat*, and plasmid-encoded enterotoxin *senB*. Finally, strain H25 D:ST-38 contained by far the most with 13 virulence factors, including *sat* as well as *aggR*, *aggA-D*, dispersin *aap*, dispersin transporter *aatA*, and *aggR*-activated regulator *aar* (Table 2).

#### Antibiotic resistance genes and mobile elements

Acquired antibiotic resistance genes and chromosomal point mutations conferring resistance were identified for all ESBL-producing *E*. *coli*. All detected resistance genes were located on one to two scaffolds per strain (Table 3). Almost all phenotypic resistances observed in disk diffusion assays could be attributed to the identified resistance determinants.

**Table 3:** Antibiotic resistance phenotype and genotype of ESBL-producing *E*. *coli* from irrigation water.

*ID*	*origin* *(PG*, *ST)*	*antibiotic resistance phenotype*	*acquired antibiotic resistance genes (grouped by contigs)*	*plasmid replicon* *(% ID; HSP/query)*	*point mutations*
H2	canal B (A, ST-361)	AM, FEP, CPD, AMC, CRO, CXM, TOB, CN, NA, NOR, SXT, K, CIP, LEV, TMP, SMZ, TEMO, KF, CAZ, CTX	*I. strA*, *strB*, *bla*_TEM-1B_, *mph(A)*, *sul2*, *dfrA14* *II. aac(3)-IIa*, *bla*_CTX-M-15_	*I.* IncY (100; 765/765) *II.* *n*.*d*.	parC p.S80I gyrA p.S83L gyrA p.D87N
H6	canal B (B2, ST-1193)	AM, FEP, CPD, CRO, CXM, NA, NOR, SXT, K, CIP, LEV, TMP, SMZ, TE, KF, CAZ, CTX	*I. strA*, *strB*, *aadA5*, *bla*_CTX-M-27_, *mph(A)*, *sul1*, *sul2*, *tet(A)*, *dfrA17*	*I.* IncFIA (99.74; 388/388) IncFIB (96.63; 682/682)	parE p.L416F parC p.S80I gyrA p.S83L gyrA p.D87N
H17	canal B (B1, ST-1079)	AM, FEP, CPD, CRO, CXM, TOB, CN, SXT, TMP, SMZ, KF, CAZ, CTX	*I. strB*, *sul2*, *dfrA14* *II. aac(3)-IId*, *bla*_CTX-M-1_	*I. n*.*d*. *II. n*.*d*.	*n*.*d*.
H10	canal C (D, ST-68)	AM, FEP, CPD, AMC, CRO, CXM, SMZ, TEMO, KF, CAZ, CTX	*I. strA*, *strB*, *bla*_TEM-1B_, *sul2* *II. bla*_CTX-M-32_	*I.* IncFII (100; 261/261) *II. n*.*d*.	*n*.*d*.
H25	canal C (D, ST-38)	AM, FEP, FOX, CPD, AMC, CRO, CXM, SXT, K, AK, TMP, SMZ, TEMO, KF, CAZ, CTX	*I. strA*, *strB*, *aphA1*, *bla*_TEM-1B_, *sul2* *II. aadA1*, *bla*_CTX-M-14b_, *dfrA1*	*I.* IncQ1^2^ (100, 529/796) *II. n*.*d*.	*n*.*d*.
H45	canal D (B1, *n*.*a*.^*1*^)	AM, FEP, CPD, AMC, CRO, CXM, ETP, KF, CAZ, CTX	*I. bla*_CTX-M-1_	*I.* IncI1 (98.59; 142/142)	*n*.*d*.
H30	spring water (D, ST-68)	AM, FEP, CPD, AMC, CRO, CXM, SMZ, TEMO, KF, CAZ, CTX	*I. strA*, *strB*, *bla*_TEM-1B_, *sul2* *II. bla*_CTX-M-32_	*I.* IncFII (100; 261/261) *II. n*.*d*.	*n*.*d*.
H22	river A (B1, ST-641)	AM, FEP, CPD, AMC, CRO, CXM, MI, SMZ, TE, KF, CAZ, CTX	*I. bla*_CTX-M-1_, *sul2*, *tet(A)*	*I.* IncI1 (98.59; 142/142)	*n*.*d*.
H40	river B (B1, ST-58)	AM, FEP, CPD, AMC, CRO, CXM, ETP, SXT, MI, K, TMP, SMZ, TE, TEMO, KF, CAZ, CTX	*I. strA*, *strB*, *aadA1*, *sul1*, *sul2*, *tet(A)*, *dfrA1* *II. bla*_CTX-M-1_, *sul2*, *tet(A)*	*I.* IncFIB (97.07; 682/682) IncFIC (95.59; 499/499) *II.* IncI1 (98.59; 142/142)	*n*.*d*.
H38	rainwater tank (c) (B2, ST-131)	AM, FEP, CPD, AMC, CRO, CXM, ETP, NA, NOR, CIP, LEV, TEMO, KF, CAZ, CTX	*I. bla*_CTX-M-15_, *mph(A)*	*I.* IncFIB (98.39; 682/682)	parE p.I529L parC p.S80I parC p.E84V gyrA p.S83L gyrA p.D87N
H44	rainwater tank (o) (A, ST-4981)	AM, FEP, CPD, CRO, CXM, NA, NOR, SXT, CIP, LEV, TMP, SMZ, F100, TE, TEMO, KF, CAZ, CTX	*I. strA*, *strB*, *bla*_TEM-1B_, *qnrS1*, *sul2*, *tet(A)*, *dfrA14* *II. bla*_CTX-M-15_	*I.* IncFIB (96.77; 682/682) *II. n*.*d*.	parC p.S80I gyrA p.S83L gyrA p.D87N

PG, phylogenetic group; ST, sequence type; % ID, percent identical bases between query and sample sequence; HSP/query, alignment length compared to query sequence length; (c), closed; (o), open; *n*.*a*., not applicable; *n*.*d*., not detected.

^1^ *adk-6*, *fumC-4*, *gyrB-14*, *icd-642*, *mdh-9*, *purA-7*, *recA-7*

^2^ truncated form of IncQ1

Resistance genes *sul1* and/or *sul2* were present in all sulfonamide-resistant strains, and trimethoprim-resistance genes *dfrA1*, *dfrA14*, and *dfrA17* were identified in six strains, all of which showed phenotypic trimethoprim-resistance. When these genes (*sul* and *dfrA*) were present in the same strain, phenotypic resistance toward antibiotic combination trimethoprim-sulfamethoxazole (SXT, Table 3) was observed. The four tetracycline-resistant strains all carried ARG *tet(A)*. Three strains carried *mph(A)* but did not show detectable erythromycin-resistance in disk diffusion assays.

With respect to aminoglycoside-resistance, the most frequently detected ARG *strA* and *strB* (also designated *aph(3”)-Ib* and *aph(6)-Id*, respectively) confer resistance to streptomycin, which was not tested in disk diffusion assays. Further, *aadA1* and *aadA5* known to confer streptomycin- and spectinomycin-resistance were detected in three strains (not tested). Strains H2 and H17 carried ARG of the *aac(3)-II* group conferring the observed gentamicin-, tobramycin-resistance, and/or kanamycin-resistance, while no ARG was found in strains H6 and H40 conferring kanamycin-resistance (Table 3). In strain H25, the observed kanamycin-resistance can be explained by the presence of *aphA1* [71].

All ESBL-producing strains carried genes of the *bla*_CTX-M_-type. Most frequent were *bla*_CTX-M-1_ (4 of 11) and *bla*_CTX-M-15_ (3 of 11) followed by *bla*_CTX-M-32_, *bla*_CTX-M-14b_, and *bla*_CTX-M-27_ (Table 3). Additionally, five strains harbored *bla*_TEM-1B_, which was never located on the same scaffold as the *bla*_CTX-M_ gene. Apart from resistance to extended-spectrum cephalosporins, ertapenem-resistance was observed in three isolates. Of note, resistance was determined based on the ECOFF value. In addition to resistance towards a broad spectrum of beta-lactams, strains H2, H6, H38, and H44 were resistant to the (fluoro)quinolones nalidixic acid, norfloxacin, ciprofloxacin, and levofloxacin and all carried chromosomal point mutations known to confer resistance. Strain H44 additionally carried the plasmid-mediated quinolone resistance gene *qnrS1* (Table 3).

**Fig 4:**
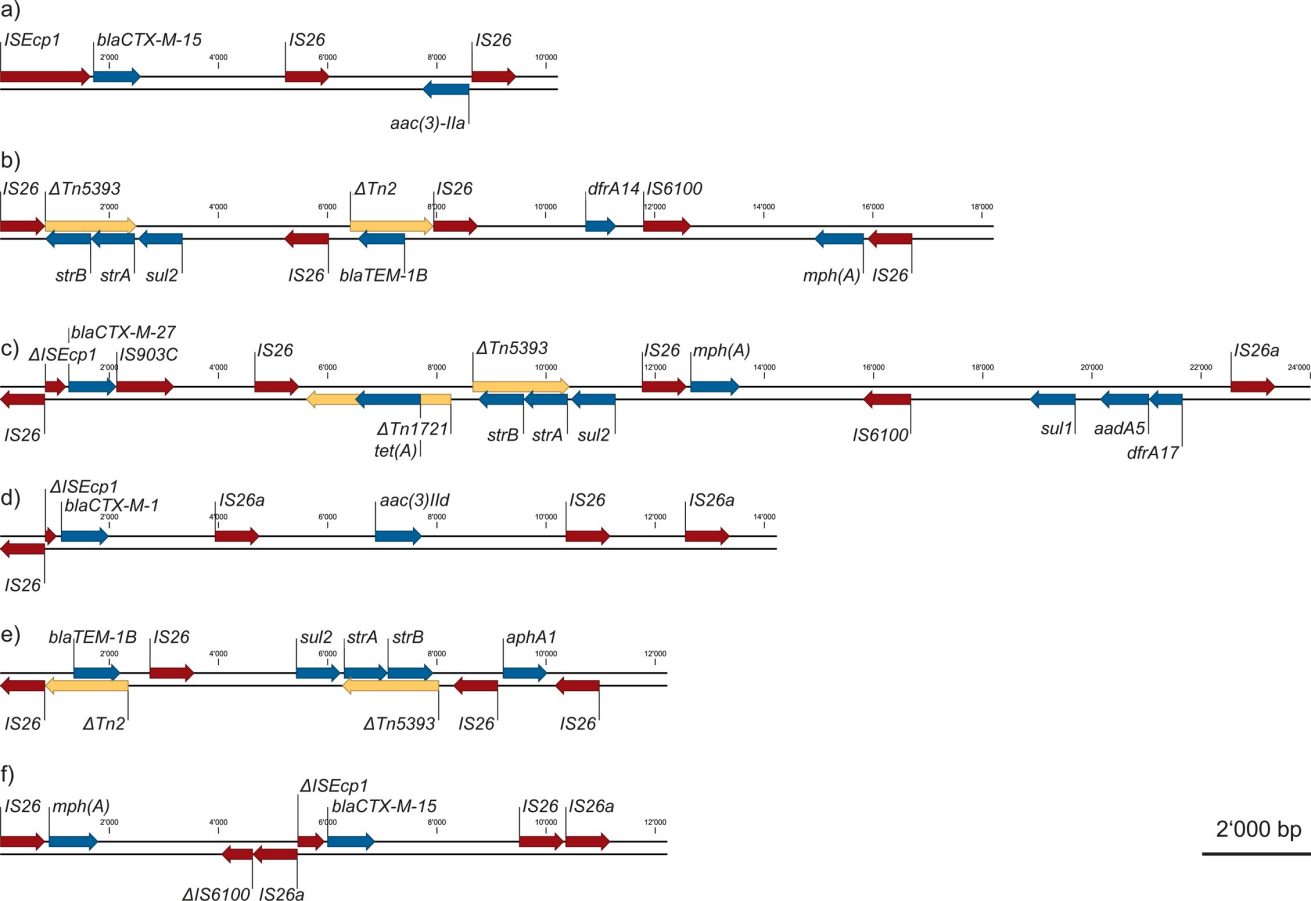
Representative ARG regions displaying ARG and associated insertion sequences. ESBL strains H2 scaffold II (a) and I (b), H6 scaffold I (c), H17 scaffold II (d), H25 scaffold I (e), and H38 scaffold I (f). IS, insertion sequence; ΔIS, partial insertion sequence; Tn, transposon; ΔTn, partial transposon. Red: insertion sequences; blue: ARG; yellow: transposons.

Apart from ARG, plasmid replicons were identified for all except one ESBL strain, suggesting mobility of the associated ARG (Table 3). Most prominent were replicons of the IncF-family, followed by IncI1, IncY, and one truncated version of IncQ1. Screening of the ARG genomic regions for other mobile elements revealed a multitude of insertion sequences and transposons, of which a representative selection is shown in Fig 4. Of special interest is the repeatedly identified insertion sequence IS26 which is frequently involved in remodeling MDR resistance plasmids [72]. ARG bracketed by IS26 or its three-nucleotide variant IS26a included *aphA1*, *aac(3)-II*a, *aac(3)-IId*, *strA*, *strB*, *mph(A)*, *bla*_TEM-1B_, *bla*_CTX-M-1_, *bla*_CTX-M-15_, *bla*_CTX-M-27_, *dfrA14*, *tet(A)*, *sul1*, and *sul2* (Fig 4). *bla*_CTX-M_ genes were additionally often associated with full-length IS*Ecp1* (representative example in Fig 4), indicating their potential to be mobilized although no plasmid replicon was assigned to almost half of them (Table 3). Finally, *bla*_TEM-1B_ was always embedded in a partial or complete Tn2, *strA* and *strB* in a partial Tn5393 (examples in Fig 4).

## Discussion

Fecal indicators *E*. *coli* and *Enterococcus* spp. have been well established for routine monitoring of water quality, and this principle has been extended to foods [44]. More recently, both *E*. *coli* and *Enterococcus* spp. have been proposed for monitoring antibiotic resistance [47]. Therefore, considering these bacteria for monitoring water quality with respect to ARB to estimate its suitability for irrigation is close at hand. Guidelines for safe use of waste- and greywater in agriculture exist [73, 74], however, they don’t include ARB, and studies investigating suitability of water for irrigation usually focus on pathogenic bacteria. Ideally, monitoring generic *E*. *coli* and *Enterococcus* spp. would yield a good prediction for presence of clinically relevant ARB and associated resistance genes. In this study, generic *E*. *coli* and *Enterococcus* spp. counts in irrigation water from different vegetable growing areas showed large variations. However, subsequent isolation of ARB strains on different antibiotics delineated a clear trend towards higher percentage of ARB-positive samples with increasing number of corresponding generic bacteria. Of special interest is the increasing number of ESBL-producing *E*. *coli*-positive water samples with increasing generic *E*. *coli* content. Whether such a correlation holds true for clinically relevant ARB of other species and related ARG remains to be studied. In any case, the presence of ESBL-producing *E*. *coli* in 22% of the investigated irrigation water samples is of great concern and emphasizes the need for monitoring irrigation water quality with respect to ARB. The cutoff for surface irrigation water presently advised by SwissGAP of 10^3^ generic *E*. *coli* per 100 ml of water [75] is not sufficiently stringent to exclude ESBL-producing *E*. *coli*-positive samples, as only 2 of the 8 positive samples in this study reached this threshold.

### ESBL-producing *E*. *coli*

#### Phylogeny and virulence factors

ESBL-producing *E*. *coli* from the different water samples were investigated in detail. Different phylogenetic groups have been assigned group-specific associations. While phylogenetic groups A and B1 encompass many commensal *E*. *coli*, groups B2 and D often contain extraintestinal pathogenic *E*. *coli* [76]. Indeed, ESBL strains of groups A and B1 harbored two to four virulence factors, while strains of groups B2 and D had four to thirteen virulence factors, including toxin-encoding genes *sat*, *vat*, and *senB* (Table 2). The sequence type/phylogenetic group combinations identified in this study have all been described before, except for strains H44 (A:ST-4981) and H45 (B1, unknown sequence type). Notably, all identified sequence types including ST-4981 have been described in association with ESBL-producing *E*. *coli* [77–84]. All these sequence types have been isolated previously from many different sources, always including humans and livestock and often water (Table 2). Based on these prior reports, humans, livestock, and wild animals (usually including birds) are all probable sources of water contamination with the detected ESBL-producing *E*. *coli*. Notably, two *E*. *coli* D:ST-68 were isolated once from spring water and once from a water canal, which have been previously isolated from humans, companion animals, livestock, and wild animals, but no isolation from water has been explicitly reported so far (Table 2).

Apart from many commensal strains, *E*. *coli* can harbor a wide variety of virulence genes and are divided into at least six main categories [85]. Pathogenic strains can be assigned a pathotype in dependence of the virulence genes they harbor. Increased serum survival (*iss*) gene, which has long been known for its role in virulence of extraintestinal pathogenic *E*. *coli* (ExPEC) [86] was detected in eight of eleven strains (Table 2). ExPEC are strains which can cause infection in organs other than the intestine, most commonly the urinary tract [85]. Other virulence factors frequently produced by uropathogenic *E*. *coli* are the type V secreted toxins vacuolating autotransporter toxin (Vat) and secreted autotransporter toxin (Sat) [87]. Encoding genes were present in three ESBL-producing *E*. *coli* in combination or alone including pandemic strain B2:ST-131 (Table 2). One strain (H6, B2:ST-1193) carried a plasmid-encoded secreted enterotoxin (*senB*) gene which has been described to play a role in development of severe diarrhea by enteroinvasive *E*. *coli* (EIEC) [88]. Apart from secreted toxins, adhesion factors play an important role for infection, both intra- and extra-intestinally. Such factors are long polar fimbrae (lpfA) as well as Iha, a putative adherence factor found in both ExPEC and diarrheagenic *E*. *coli* [89]. Encoding genes were detected in six and three strains, respectively. Finally, ESBL-producing strain H25 D:ST-38 outnumbered all other isolated ESBL-producing strains with thirteen virulence factors. Sequence type 38 has been associated with enteroaggregative *E*. *coli* (EAEC) [81]. Indeed, strain H25 harbored known EAEC virulence genes such as dispersin *aap* [90] or *aggA* and *aggR* [91]. The latter two genes have been suggested to suffice alone or in combination for identification of pathogenic EAEC strains. The presence of a pathogenic EAEC strain resistant to extended-spectrum beta-lactams in irrigation water used for fresh produce emphasizes the need for monitoring and regulating irrigation water quality.

#### Resistance phenotype and genetic background

ESBL-producing *Enterobacteriaceae* such as *E*. *coli* as well as vancomycin-resistant *Enterococcus* spp. (VRE) are among the clinically most relevant ARB. Being both typical inhabitants of the intestine and occasionally notorious MDR pathogens, they have the potential of establishing in the human gut where they can spread ARG or cause disease. In fact, 5.8% of the Swiss healthy population have been estimated to carry ESBL-producing *E*. *coli* [92]. We detected such strains in 22% of water samples all used for fresh produce irrigation, while no VRE were isolated. Presence of ESBL-producing *Enterobacteriaceae* in surface waters has been described previously [31]. The proportion, however, of ESBL-positive water used in vegetable growing areas for irrigation was unknown and seems considerable. On the other hand, VRE have been detected in wastewaters including un-chlorinated effluent but not water used for irrigation, although increased future use of reclaimed waters for irrigation has been predicted [93]. Genetic analysis of eleven ESBL-producing *E*. *coli* showed that apart from a variety of virulence factors, they harbored a high diversity of ARG, always including one gene of the *bla*_CTX-M_-type. Concurrent resistance towards fluoroquinolones and various other antibiotic classes considerably narrows down available treatment options. Including the fact that most detected ARG were situated on a plasmid backbone and/or associated with mobile genetic elements, such bacteria in water must be eliminated before applying it for irrigation. From the detected resistance plasmids, IncFII and IncI1 are considered epidemic having the highest occurrence among typed plasmids [94]. The IncF family in particular–the most frequent replicon family in this study–is detected in a variety of *Enterobacteriaceae* within which they can spread. The frequently detected IS26 (full-length, on 12 of 18 scaffolds) has recently been designated as major player in MDR plasmid remodeling [72] and IS*Ecp1* has been associated with genetic mobilization of *bla*_CTX-M_ [95].

Antibiotic resistance profiling using disk diffusion assays is routinely performed in clinics for resistance monitoring and determination of appropriate treatment strategies. With the racing development of next generation sequencing technologies, plug-and-play tools for *in silico* detection of ARG based on sequence data have been developed, envisioning clinical decision-making based on molecular data rather than phenotypic tests. Our data showed almost perfect concordance between phenotypic and genotypic resistance profiles. Nevertheless, a few phenotypic resistances could not be explained based on the detected genetic determinants. Also, certain treatment options might be missed as the example of *mph(A)* shows, where despite presence of the ARG no phenotypic resistance was observed. Thus, the molecular approach appears to result in a very accurate albeit imperfect prediction of phenotypic resistance.

### Conclusions

We could show high occurrence of ESBL-producing *E*. *coli* in irrigation water, with one positive sample in five. We further showed that these strains harbor a diversity of mobile ARG and a variety of virulence factor genes, including toxin genes. To determine the amount of such bacteria in irrigation water, a quantitative rather than enrichment-based approach is needed. However, whatever their abundance in the water, their spread via irrigation onto foods which are consumed raw poses a potential health risk which must be avoided. Therefore, monitoring and regulating irrigation water quality as well as developing affordable sanitation technologies is crucial, especially as the use of surface water and reclaimed wastewaters tends to become more and more common agricultural practice.

## Supporting information

S1 Fig Aerobic mesophilic counts (AMC) versus fecal indicator counts. Colony forming units (CFU) per 100 ml of water are indicated. Dotted line marks the limit of detection. No significant correlation was observed (ns, not significant).
(TIF)

S1 File SPAdes results. Fasta files containing scaffolds generated from ESBL-producing *E*. *coli* sequencing data using SPAdes algorithm.
(ZIP)

S2 File BWA-SGA results. Fasta files containing scaffolds generated from ESBL-producing *E*. *coli* sequencing data using SGA scaffolder.
(ZIP)

S3 File MARA sequences. Fasta files containing scaffolds generated from ESBL-producing *E*. *coli* sequencing data and selected for uploaded to online tool MARA.
(ZIP)
